# Baggage scanners and their use as an imaging resource in mass fatality incidents

**DOI:** 10.1007/s00414-019-02132-y

**Published:** 2019-08-08

**Authors:** Genevra D’Arcy, Nicholas Márquez-Grant, David W. Lane

**Affiliations:** Cranfield Forensic Institute, Defence Academy of the United Kingdom, Cranfield University, Shrivenham, SN6 8LA UK

**Keywords:** Disaster victim identification, Forensic anthropology, Radiology, Mass fatalities, X-rays, Baggage scanners

## Abstract

Disaster victim identification following a mass fatality incident is focussed on identifying the deceased and returning them to their families as quickly as possible, while gathering as much information as practical to aid investigators in establishing the cause of the incident. Ante-mortem data is gathered and compared with the post-mortem data obtained in order to positively identify the deceased. This paper presents results from a study concerned with the first part of the process of identifying the deceased—the triage or Primary Survey and how this can be done without access to hospital facilities such as conventional X-ray imaging or computed tomography. In particular, this study focuses on the imaging undertaken prior to the opening of the body bag by a multidisciplinary team, and how this imaging can assist particularly when forensic anthropologists are involved in the identification process. There are several advantages to imaging the body bags before they are opened and one of the most important is safety. Thus, this paper examines the viability of using a baggage scanner as a practical resource for X-ray imaging, as many regions worldwide may not be able to access conventional imaging equipment. Baggage scanners are readily available and found in airports and various government buildings. The baggage scanner is particularly suited to this task and produces images that can be used by forensic anthropologists to distinguish between human and non-human remains, identify items of evidence and personal effects, and even perform a preliminary or partial biological profile. When considering their response plans, emergency responders should consider including baggage scanners as a contingency for screening body bags if no other imaging system is available.

**Electronic supplementary material:**

The online version of this article (10.1007/s00414-019-02132-y) contains supplementary material, which is available to authorized users.

## Introduction

A mass fatality incident (MFI) can be a natural or human-made disaster, resulting in a number of fatalities that exceed the local resources available to deal with them [1]. The size of a MFI is variable, for example a small number of deaths may overwhelm one country or region but fall within the realms of normality for another, and the response capability is dependent on the country or local infrastructure, skill level, and the equipment available [2, 3]. Identifying the deceased in these types of incidents, a process termed disaster victim identification (DVI), involves a multidisciplinary team that performs the recovery of remains, the gathering of ante-mortem data, analysis in the mortuary, support to relatives, and the repatriation of the human remains [4]. In these incidents, the human remains may either be intact, fragmented, burned, commingled, distorted, and in various stages of decomposition [5]. All these factors make the identification of the deceased a long and challenging procedure [e.g. see 4, 6]. This study, in particular, is aimed at the role of the forensic anthropologist in this process and the use of an imaging method not previously or routinely used: the airport baggage or security scanner that may be used for screening when conventional radiographic facilities are not available.

The forensic anthropologist, in their role as an expert in human skeletal remains [e.g. see 7, 8], can provide valuable assistance at the scene in identifying the presence of bone fragments in various conditions that may otherwise be unrecognisable to the untrained eye, and distinguishing between human and non-human bone [5, 9, 10]. Once in the mortuary setting, the forensic anthropologist may work in tandem with the forensic pathologist and forensic odontologist, amongst other specialists both medical and legal, in order to estimate age, sex, stature, and ancestry, and examine unique identifying features that may aid in the identification of the deceased. In addition, the forensic anthropologist may analyse evidence of trauma in order to provide investigators with a possible sequence and reconstruction of events [11, 12].

### The role of radiology in MFIs

In a mass fatality incident, the role of radiology is three-fold: *safety* (detection and location of dangerous objects within the body bag); assisting the pathologist in *determining cause of death* (injuries, foreign bodies, and evidence location); and assisting with *identification* (personal effects, dental fragments, estimation of age, sex, stature, and ancestry, comparison of ante- and post-mortem information). Where radiology is available, body bags prior to opening in the mortuary are put through X-ray scanning in a process termed ‘triage’ in which the material inside can be visually sorted into unsafe or safe, human or non-human, and whole or fragmented [4, 13, 14]. The experience gained from large-scale mass fatality incidents worldwide has highlighted the need to have this imaging done at the scene within the Major Incident Mortuary [14]. While the forensic anthropologist can do a more accurate job of identifying skeletal remains by direct observation, radiographic examination of fully or partially fleshed remains is useful when there are no facilities to deflesh or macerate the bones [15]. For example, radiographs of the deceased have been crucial in the identification of some individuals when personal effects, not found in autopsy or through manual search, were observed still attached to badly burned bodies in the X-ray image [16]. In the Oklahoma City bombing in 1995, six victims were identified exclusively through features found on radiographs and compared with ante-mortem records, and in many cases, they were crucial in locating evidence [17]. Moreover, in the identification process for the victims of the attack on the Pentagon on 9/11, radiology guided all subsequent examinations as it revealed the state of the remains, what was or was not present, personal effects, and features that assisted in age estimation [18]. Kahana and Hiss describe several examples including a plane crash, ship collision, and suicide bombings in which radiographs have been successfully used to identify fragmented remains impossible to identify by other means [19].

A C-Arm fluoroscope is generally used for the Primary Survey triage of whole bodies as it is a fast, real-time observation of the body bag and can identify sharp objects, ballistics, and personal effects amongst the body and debris [20]. Digital radiography can be used for Primary, Secondary, and Tertiary Surveys for specific body parts such as dentition, unique ante-mortem features (healed fractures, pathological conditions, implants), and any trauma as a result of the incident [16]. Computed tomography (CT) scanners not only provide important 2D images, but also a very detailed and high-quality 3D image that can be used for all three surveys, replacing other imaging methods, and can even be used instead of a physical autopsy [20, 21]. However, CT scanners are expensive, and lack of training and experience has been a point of concern for its use in a forensic setting [20, 21]. Multidetector CT (MDCT) is ideal for identifying fragments of metal important to the investigation as well as highlighting unknown injuries and are particularly useful when the remains consist of whole bodies or large body parts [22]. There have been a number of investigations of mass fatalities in recent years using CT scanners [23–26], and they are now included as standard practice in some mortuaries around the world and have become an integral part of the post-mortem imaging and identification process [27–30].

### Baggage/security scanners

Baggage scanners are found at airports worldwide, customs, and government buildings such as court houses and other security-critical buildings, so they are often readily available and require very little operator training. They work in a similar way to conventional medical X-rays in that an X-ray beam is directed at the object (usually a suitcase, briefcase, parcel etc.) and those that pass through the object hit a detector and are converted into an image that can be seen by the operator [31]. Unlike medical X-rays, baggage/security scanners create the image by mechanically passing the object through a fan-shaped X-ray beam that is directed onto a linear detector array that builds up the image. This detector is constructed in two layers to distinguish low-energy and high-energy X-rays which are used to determine the average atomic number of the material and differentiate different classes of material. Images can be presented either in black and white or in colour: the colour depends on the effective atomic number (*Z*_eff_) of the material at each pixel location and is recognised as organic, inorganic, or metallic [31]. All manufacturers use shades of orange to identify organic material (*Z*_eff_ < 11), mainly because explosives are made of organic materials and these are the primary concern of the operators [32]. Shades of blue (or purple) identify heavier elements (*Z*_eff_ > 18) such as iron, steel, gold, and silver. Between these ranges (11 < *Z*_eff_ > 18), shades of green identify materials such as some plastics, glass, aluminium, and cooking salt, while areas of the image that are impenetrable to X-rays show up black [33].

During the Persian Gulf War in 1992, the Dover Air Force Base mortuary in Delaware, USA (it had been set up during the Canary Island crash in 1977), was fitted with airport baggage scanners. These were used to scan remains arriving from the Persian Gulf for live ammunition as well as large amounts of debris that had the potential to contain human remains and other evidence [16]. A study by Goodman and Edelson alluded to a suggestion made that security (baggage) scanners be used in the temporary mortuary for the Primary Survey, but it is unclear whether the suggestion was made by the authors or by the participants of the identification process at the time of the crash. The study used security (baggage) scanners to image body bags containing fragmented human remains and other debris. The contents were identified, and the efficiency of this method compared with the time taken and accuracy of contents identified in a manual search [34]. Another study done in 2006 by a Spanish team of medical forensic specialists looked at how good a baggage scanner image of cadavers was with regard to identifying bone, trauma, personal effects, etc. [35]. They used human cadavers, both whole bodies and fragmented ones, with different post-mortem intervals in different stages of decomposition and different causes of death. Both these studies [34, 35] concluded that this method of imaging is fast and allows the viewer to easily distinguish between human and non-human materials by means of the colour image produced; that the machine is readily available; and that much of the bone structure can be seen. In addition, they also agree that this method should always be followed up with additional imaging using conventional X-ray or manual inspection by the relevant specialists such as pathologists, odontologists, and anthropologists [34, 35]. These are the only two published studies to our knowledge that specifically use a security (baggage) scanner to image human remains. The remoteness of some regions worldwide as well as the lack of advanced skill level, resources, and equipment may see this imaging technique as valuable. In some low- and middle-income countries (LMIC), the equipment is also often donated by organisations such as the UN (United Nations) and other INGOs (International Non-governmental Organisations), with initial training in its use and maintenance [36]. For these reasons, this research is important and necessary: local equipment and level of skill cannot always be relied on, the geography of remote locations is not always amenable to bringing large and sensitive equipment in, and there are airports containing baggage scanners in nearly every country [37] that can be pressed into service.

This study used pig remains, archaeological, and anatomical human skeletal remains, and scanned them in a baggage scanner. The resulting images were examined in order to provide answers to several questions about the viability of using such a machine in a MFI scenario, as well as highlighting any limitations with the method of imaging.

### Aims of the study

The overall aim of this research was to investigate the viability of using such a common airport baggage scanner to triage human remains in a MFI scenario. More specifically, this study was aimed at determining, from a forensic anthropological perspective, if the images obtained from the baggage scanner were clear and detailed enough to:

- Identify skeletal elements.
- Identify whether body parts of more than one individual are present.
- Distinguish between human and non-human (animal) skeletal remains.
- Distinguish juvenile from adult bones by examining degree of skeletal maturity.
- Perform a biological profile where possible in which age, sex, stature, and ancestry can be estimated. This included assessing an appropriate method to measure long bone length.
- Identify pathological alterations in bones.
- Observe the presence of any unique identifying features that may require further radiographic investigation.
- Understand how different types of soft tissue and skeletal remains (fresh, dry, cremated) may be observed.
- Identify and locate any hazardous material such as sharps or glass.
- Identify the location of any projectile fragments with possible associated bone injury.
- Observe the location of personal effects

In addition, a number of figures were sent to forensic anthropology practitioners for an independent assessment.

## Materials and Methods

Two types of samples were examined during the study: firstly, standard test pieces (STP) and secondly, human and non-human remains and associated artefacts contained in body bags. The STPs are standard objects used in industry for quality assurance and in this study were used to establish resolution and areas of image distortion.

Items scanned included porcine (*Sus scrofa domesticus*) tissue: trotters, fleshed ribs, defleshed long bones, scapulae and sections of spine. These were obtained from a local butcher and all from animals slaughtered according to UK accepted standards and protocols. Two of the pig trotters were frozen for several days then defrosted on the day of scanning, and two were bought on the day of scanning and were not frozen at any point. Animal (non-human) remains were used as it was not possible to gain access to fleshed human remains in the time frame of the research and for a number of ethical reasons. Also used were nineteenth-century archaeological and anatomical human bones curated at Cranfield Forensic Institute, Cranfield University, bullets and casings held by the university, and personal items of the first author (GD) such as a wallet, phone, pen, medication, syringes and needles, glass fragments, and clothing items. Natural debris in the form of sticks, branches, and leaves, obtained from the university grounds, were included to simulate some of the sort of debris that may be present along with any human remains and put in the body bags. Body bags were carefully loaded with a range of different materials according to several different scenarios and were supported on thin wooden boards to reduce the likelihood of the different objects moving as the bag was placed on the conveyor belt. A wire mesh of the type used for small animal enclosures (rabbits, guinea pigs, etc.) was used as a measurement scale to experiment with measuring the bones.

Once all the scanning was completed, a questionnaire was sent to several (*n* = 12) forensic anthropologists from different countries and with various levels of practical experience (between 5 and 25 years), in order to assess the usefulness of the images. Each observer was sent a set of images and was asked to describe what they could observe (see Online Resource 1).

### Baggage scanner

The baggage scanner used was a Smiths Heimann Systems Hi-Scan 7555i X-ray inspection system with a tunnel opening of 755 mm wide by 555 mm high. The conveyor belt speed was 0.2/0.24 ms^−1^ and can manage an evenly distributed load of 160 kg. The unit is a dual-energy X-ray system operating at 140 kVp that directs its beam diagonally across the tunnel onto an L-shaped detector. Images were generated in black & white as well as in false colour for material discrimination using the proprietary HI-MAT system and were displayed on flat panel LCD monitors. This type of baggage scanner is part of Smiths Detection’s current product range (as noted in June 2019) and is typical of the type of scanner found in many airports, government buildings, prisons, and sports/entertainment venues. Correct operation of the scanner was confirmed by running the STPs (IEEE ANSI N42.47-2010), which consisted of square plates with rows of ball bearings or wires in increasing sizes and thicknesses.

### Scanning process

Each STP was put through the scanner in three positions: on the left-hand side of the belt, in the middle, and on the right-hand side of the belt. Four scans were undertaken in each placement, changing the orientation of the STP for each scan to allow distortion of the image to be observed from all four sides of the plate. The distortion is caused by the angle of the X-ray beam that scans the object passing through the tunnel and the shape of the detector arrays; therefore, these industry tests were important in guiding the operator in placing the object to be scanned in the optimal position on the belt to minimise the effects of image distortion.

**Fig. 1:**
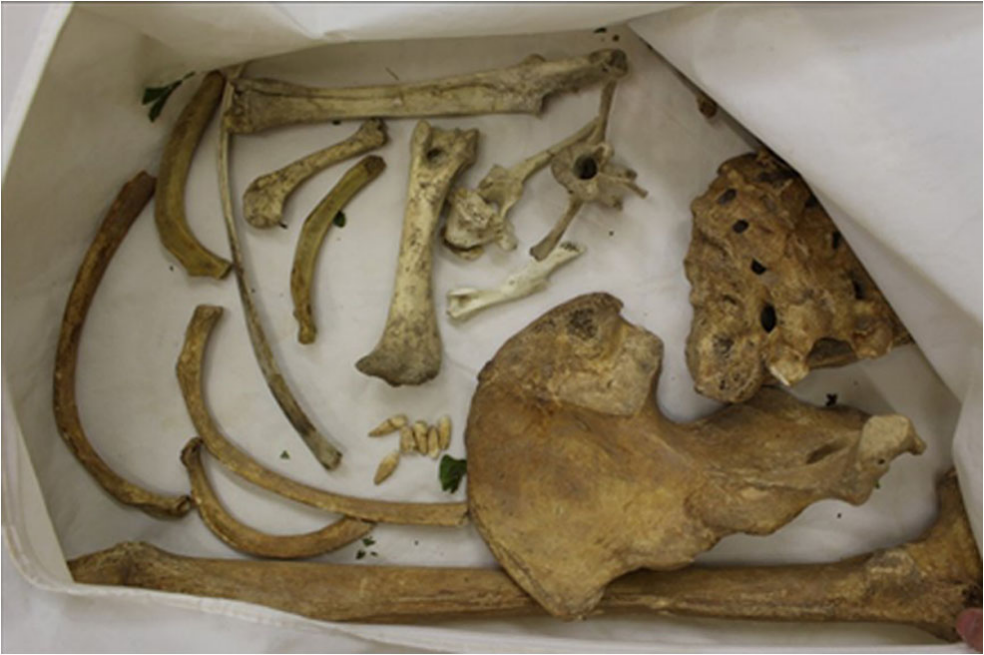
Commingled animal and human bones. The deliberate placement of bones in the body bags was done so that the viability of the method could be studied initially

Details of each scan are presented in Online Resource 2 with the pig tissue, archaeological specimens, and other items scanned in pre-set groupings. For the first scanning session, pigs’ trotters wrapped in cling film, simulated wet bone scenarios and were commingled with the bullets, casings, natural debris, and personal effects inside a body bag. The same was done with the archaeological and anatomical human remains that represented dry bone scenarios. A pig’s trotter was charred in a fast-burning fire for 15 min before being scanned, and archaeological cremated bone samples were scanned to explore whether or not cremated bone could be identified. Animal and human bones, as well as commingled human bones, were grouped together as the human vs non-human bone identification is often the first question forensic anthropologists have to answer (Fig. 1). Bones showing ante-mortem trauma were scanned to see if the healed fractures could be observed, and bones evidencing pathological conditions (e.g. degenerative joint disease on vertebral bodies) were also scanned for their ability to be seen. The body bags were placed on a wooden tray to help avoid unnecessary movement of the bag as it passed through the curtains at the tunnel entrance, as this would result in a stretched and distorted image.

The second scanning session focussed on looking at specific aspects such as age-at-death, sex, and stature estimation. Juvenile human bones were scanned to establish if they could be identified with their unfused epiphyses; fleshed pig tissue in the form of sections of adult ribs and spine were included to explore how the surrounding flesh affected the detail of the underlying bone. In addition, defleshed animal long bones were scanned to study the difference, if any, between wet and dry bone in the images; and human pelvic bones of different ages were scanned to see if the pubic symphyses could be seen clear enough for age-at-death estimations. These were placed in a cardboard box instead of a body bag as it had already been established in the first session that the body bag would not interfere with the image.

Finally, part of the biological profile forensic anthropologists perform on human remains is the estimation of stature. This is done by measuring the length of a long bone, preferably the femur but other long bones can also be used [38–40]. The model of baggage scanner employed in this study did not have the facility to take measurements from scanned objects. This necessitated improvised measurements such as could be used by any anthropologist. In this study, one human femur with a maximum length of 420 mm as measured by an osteometric board was employed. Two methods of measuring this long bone from the images produced were tested. The first used the wooden tray on which the remains were scanned: this was measured and became the object of known size, which was then scanned, the image measured and scaled accordingly to convert it to a physical length. The second method used the wire mesh (as used in small animal enclosures, although a steel barbeque grill or rebar could also be employed) acting as a grid system: each square in the mesh was measured to be 12.7 mm wide. The number of squares from one end of the bone to the other was counted and thus converted to a physical length.

## Results

### Images

**Fig. 2:**
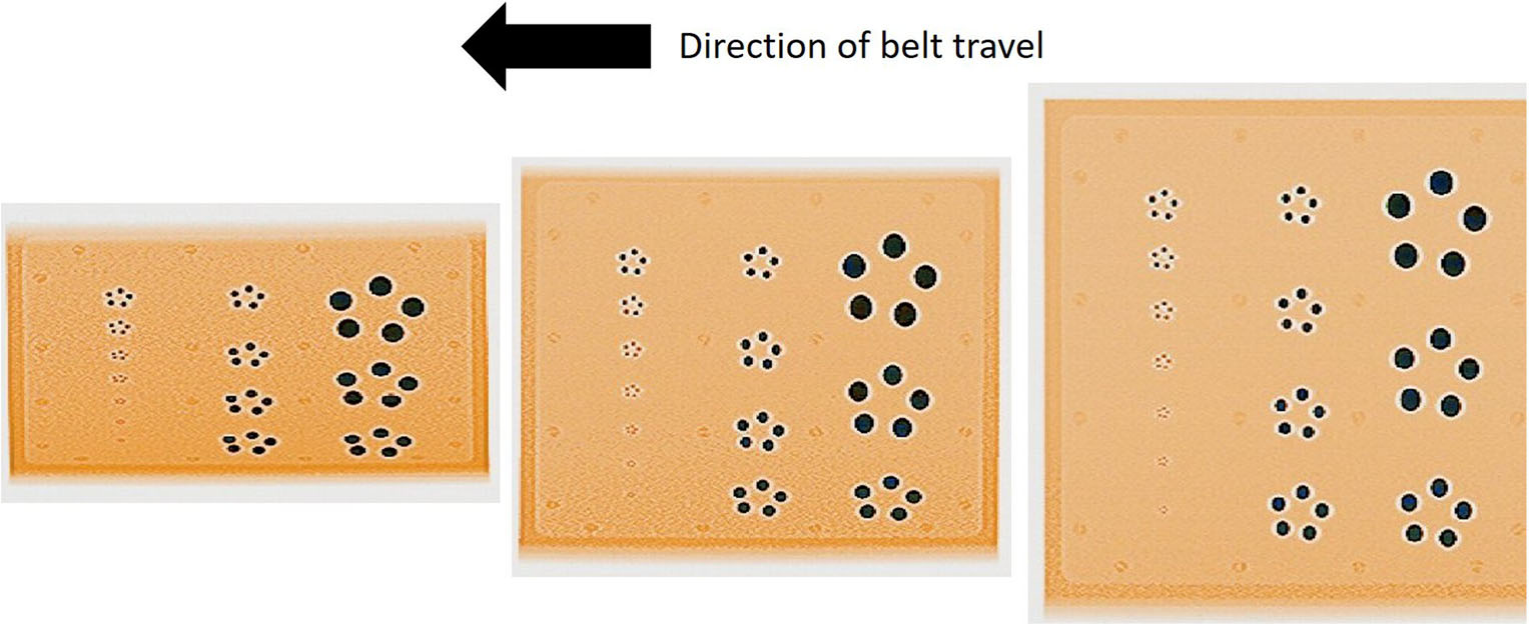
Images of the IEEE ANSI N42.47-2010 ball bearing STP when positioned on the left-hand side of the belt (left), centre of the belt (centre), and right-hand side of the belt (right) when viewing the belt at the entrance of the scanner

The STPs showed very clearly that the distortion in the image was most prominent along the bottom edge of the image, which would be the left-hand side of the belt at the tunnel entrance. The distortion was less along the top edge of the image (right-hand side of the belt) and the central band appeared distortion-free (Fig. 2). While the body bags could then be placed centrally, the contents would not necessarily pass through the centre only but could overlap into the areas of distortion. This would need to be accounted for in any size calculations done as discussed later.

**Fig. 3:**
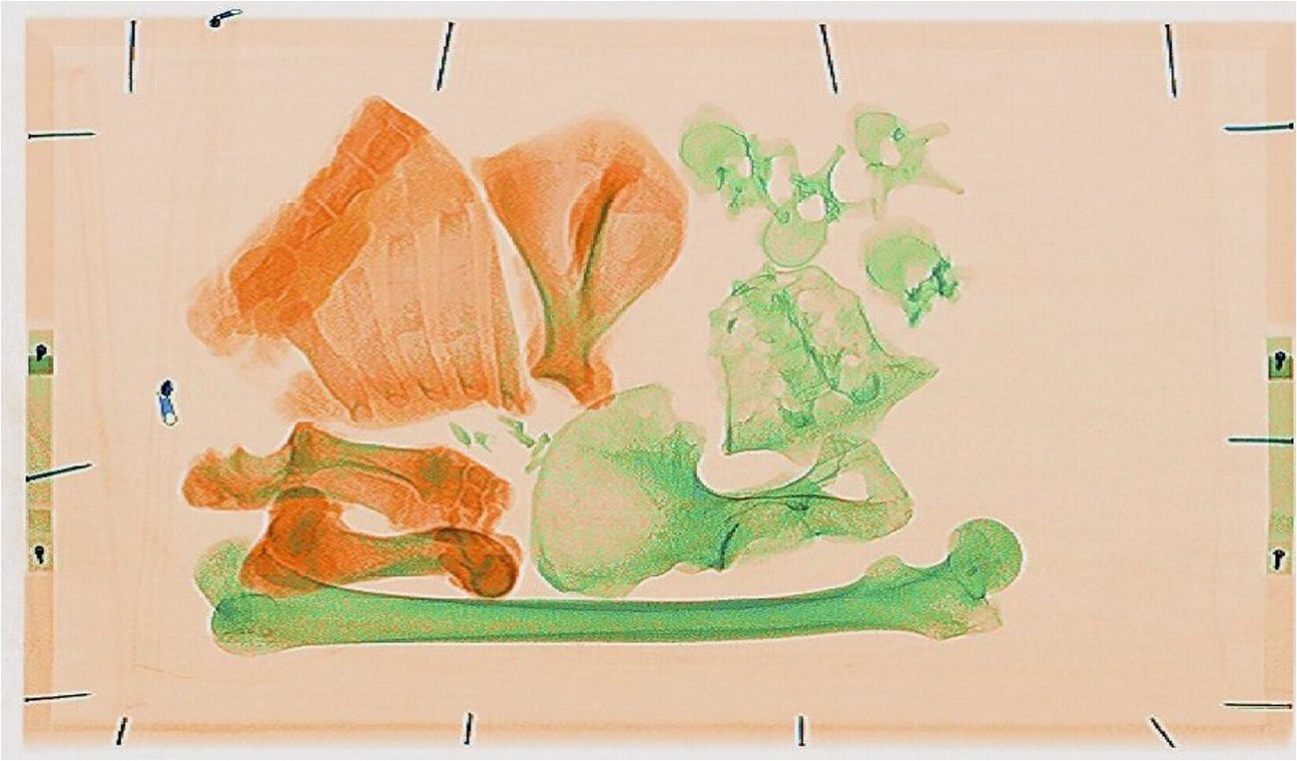
Fleshed and newly defleshed animal bones (orange) and dry human bones (green). The difference in colour pertains to the water content of the bones rather than whether they are animal or human

**Fig. 4:**
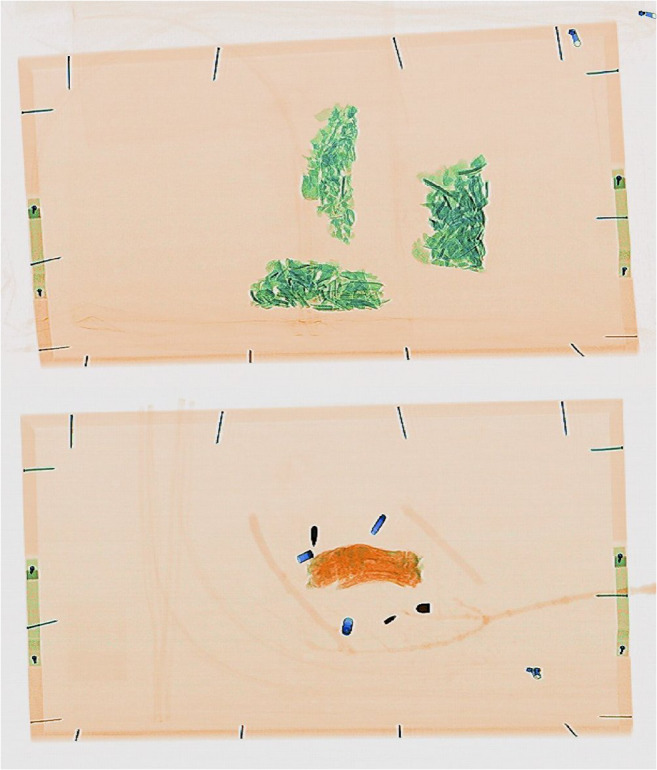
Cremated human bone (top) and a charred pig trotter with bullets, casings, and tree debris (bottom). The thin branches and leaves from the tree are barely visible, drawing the focus to the metal objects and flesh/bone

The images of the pig tissue, human bones, natural debris, and personal effects showed clear differences in colour making it easy to distinguish between fleshed remains, bone, and more general objects at first glance. As can be seen from Online Resource 2, fleshed and newly defleshed bones (wet bones), sticks/branches, plastic pens, and syringes are all organic materials and so were indicated in an orange colour, while the dry bone (archaeological and anatomical specimens), being mostly inorganic, were indicated in a green colour (Fig. 3). The cremated bone indicated in a slightly duller, darker green, although the charred pig trotter was indicated in an orange colour despite the organic tissue being burned (Fig. 4). Metal objects such as the bullets, casings, the body bag zip, nails in the wooden tray, keys, parts of the pen, and coins in the wallet all showed in shades of blue according to their higher *Z*_eff_. For example, the bullets were so dark a blue that they were nearly black because they were largely impenetrable to X-rays, while the casings were a lighter blue (Fig. 4) and the leaves did not show up in the image at all. Pigs’ trotters that had been frozen and then defrosted showed no difference in the scan images when compared to the unfrozen pigs’ trotters.

**Fig. 5:**
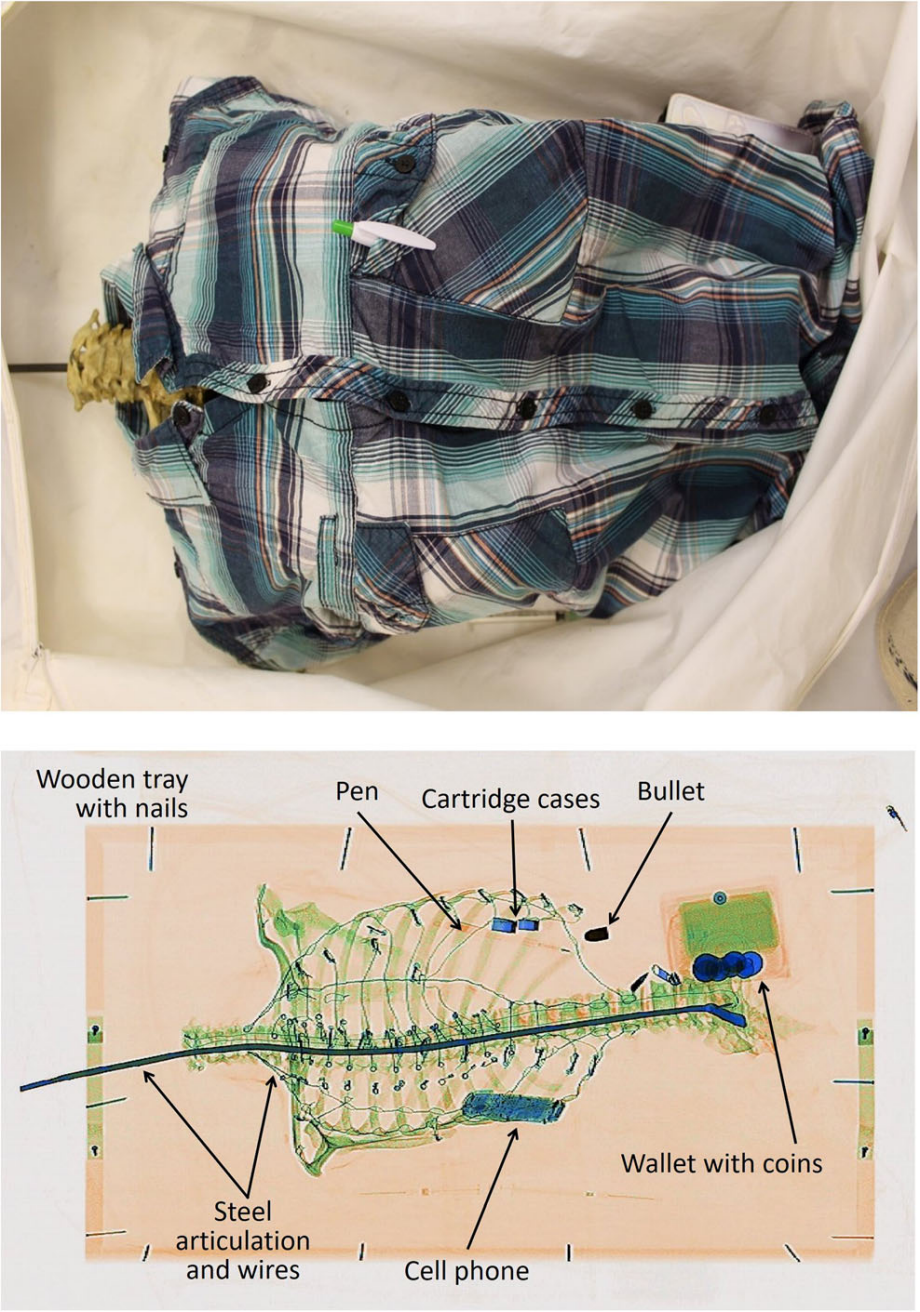
Photograph of clothed, articulated torso with personal effects in the pockets (top), scanner image of the articulated torso with clothing and personal effects visible (bottom). The torso bones were articulated using a metal rod, wire, and screws which are visible in the scanner image. The clothing is barely seen, allowing the observer to immediately see the bones and personal effects, including hazardous items such as bullets

**Fig. 6:**
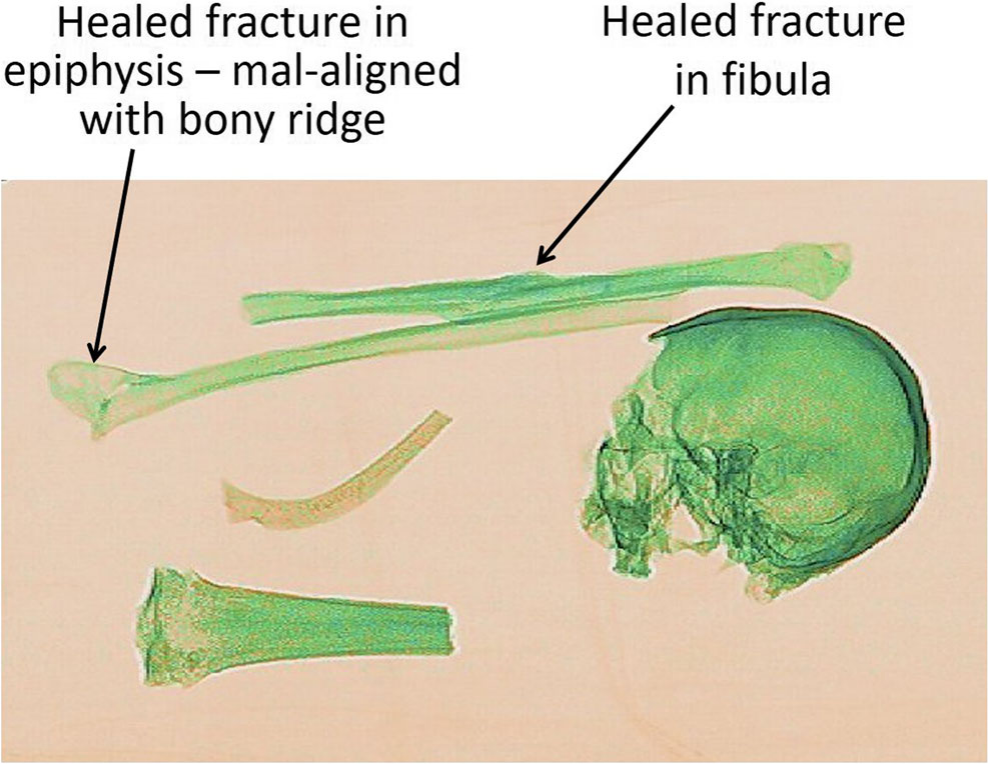
Bones exhibiting healed trauma. The image quality does not allow for zooming in to see detail and the overlapping bones in the skull make the trauma to the skull indistinct

**Fig. 7:**
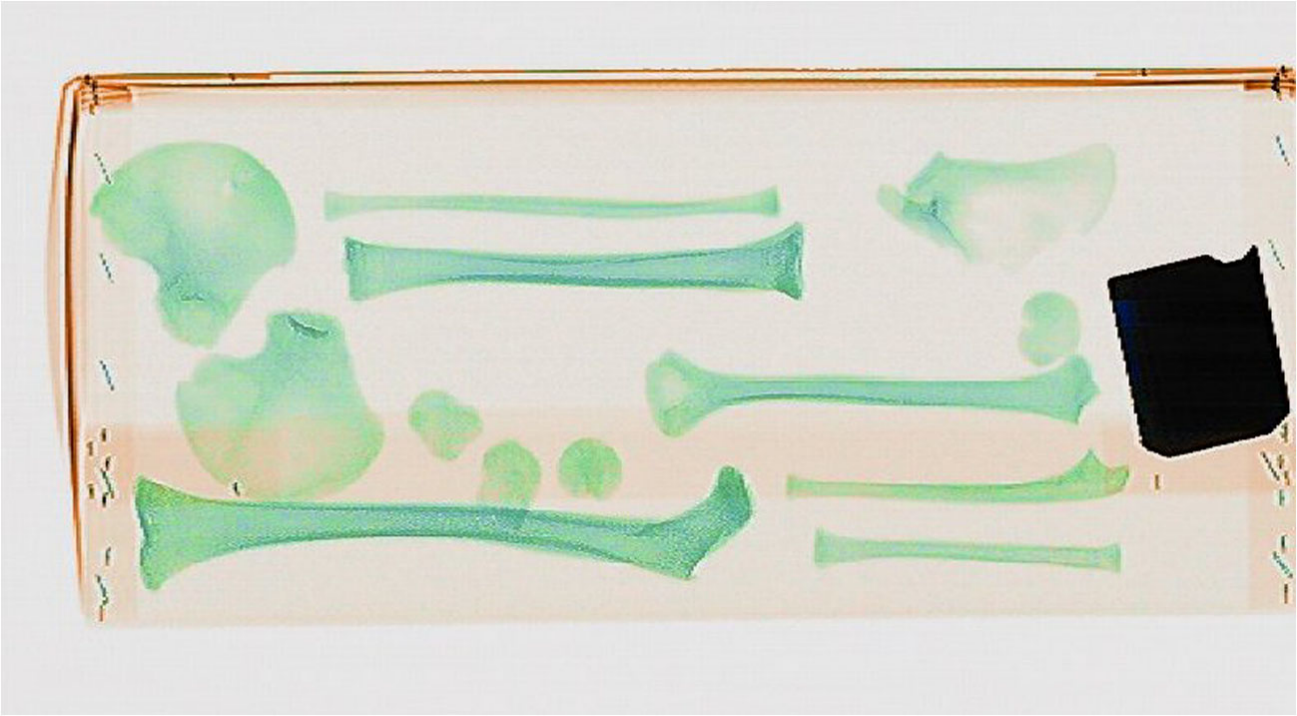
Juvenile bones showing darker green growth plates and the incomplete shapes of each bone. Note the black area is a lead block to weigh down the container during its transit through the scanner

An articulated wired skeleton (torso) dressed in a shirt with personal effects placed in the pockets (Fig. 5a) showed good detail of the spine and ribs without any interference from the clothing and personal effects, allowing examination of these bones for any trauma or identifying features (Fig. 5b). In this image, the clothing was presented as a very faint orange and could barely be seen and the bullets and casings again appeared as dark and light blue respectively. In Fig. 6, ante-mortem trauma (healed fractures) can be observed on both fibulae. For those bones exhibiting pathological alteration, the lesions on the bone were also visible to a certain degree in the images. Juvenile bones were imaged as well, and the growth plates were visible as a darker green compared with the rest of the bone. The incompleteness of the shape of the bones was also evident (Fig. 7). In a scan of commingled human remains, bones from more than one individual were identified and a scan of human and animal bones showed clear differences in size and shape.

**Fig. 8:**
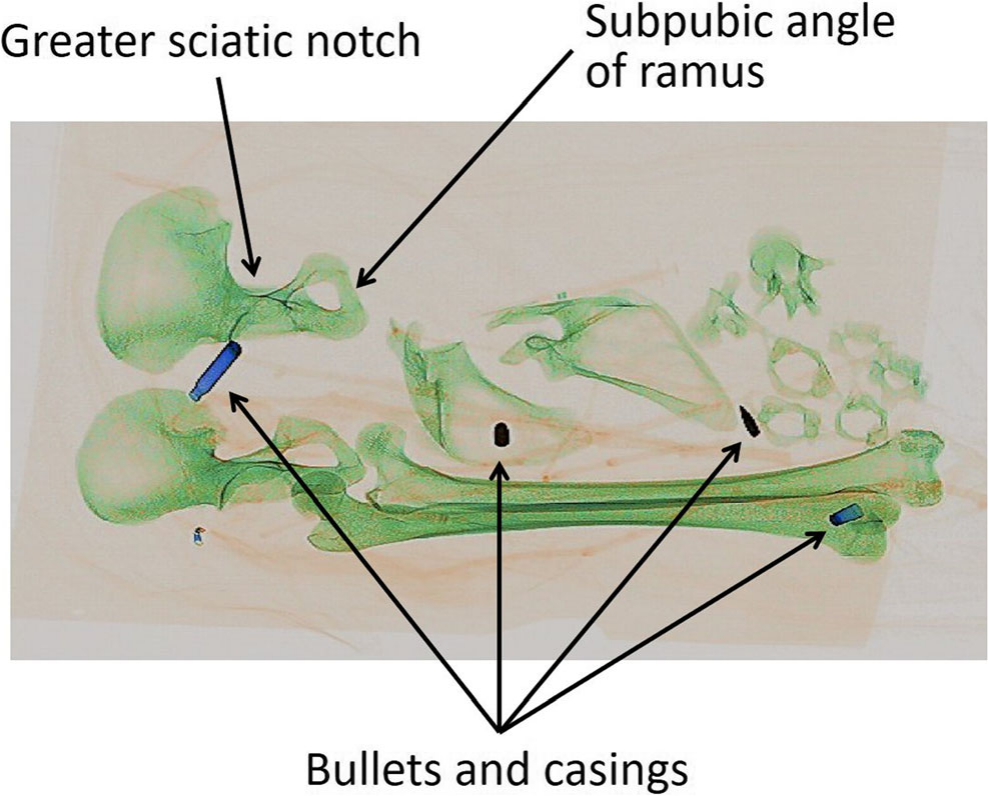
The pelvic bones showing some features that can be used for sex estimation, and potentially hazardous metal artefacts

With regard to the human pelvic bones (ossa coxa), while it was easy to see the pubic symphyses, the scanner image is not clear enough to see the detail needed to assign a phase as per Suchey-Brooks [see 38] for the age-at-death estimation. Under certain circumstances, the orientation of the human remains may allow for sex estimation. For example, features used to establish sex can also be seen in the pelvic bones such as the greater sciatic notch and the subpubic angle (Fig. 8). Therefore, in this instance, sex estimation of the individual may be possible using these images.

Online Resource 3 summarises the results of the questionnaire sent to the 12 anonymous observers. Out of the ten images presented, all the human bones were identified by all twelve observers except for image 4 that contained a large number of bones with some lying close to others: in this image, only 17% of the observers identified all the bones, while the other 83% of observers missed between 1 and 7 of the bones in their list. All observers distinguished correctly between human and non-human (animal) bones in the images. The glass fragments were identified as possible skull fragments by 17% of the observers. The cremated bone fragments were identified as such by 33% of the observers, with another 42% describing them only as bone fragments or human remains. There was a varied response to the questionnaire, but for the most part, the identification of the type of human bones was accurate, as was the distinction between human and non-human bones, and between bone and personal effects.

### Bone measurements

**Fig. 9:**
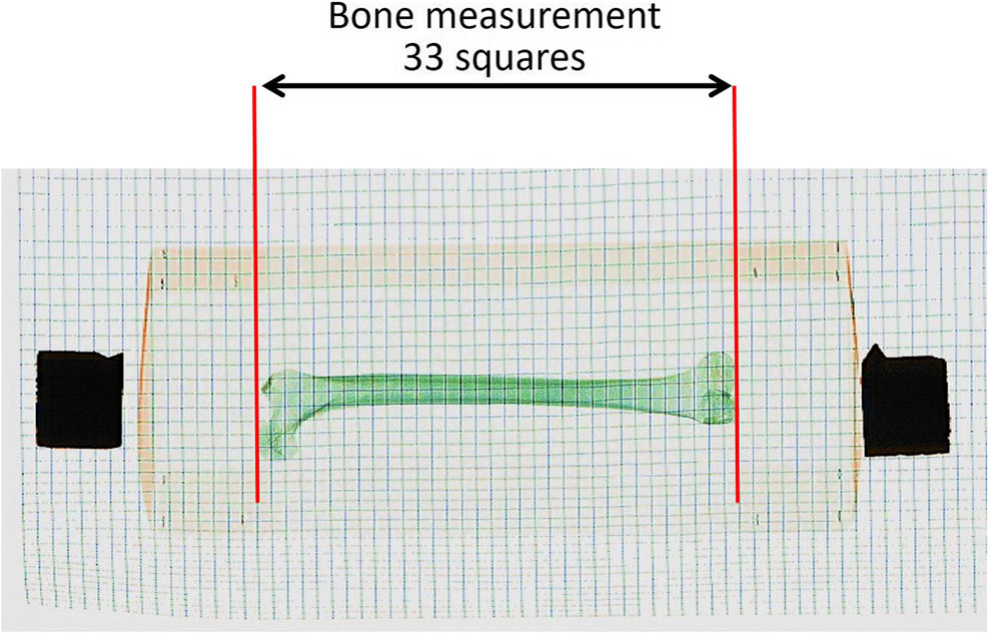
Image for bone measurement: the femur was placed on top of the wire mesh used as a grid system. The squares (of known size) were then counted and the length of the bone calculated

Because some baggage scanners do not have a scale on the resulting images, measuring the length of long bones for stature estimation would have to be determined by taking physical measurements from the screen. In this study, a human femur with a maximum length of 420 mm was employed. The wooden tray method resulted in a difference of ± 1.5 mm between the image measurement and the actual measurement; and the wire mesh method (Fig. 9) gave a difference of ± 1.0 mm between the image and the actual length of the femur. This indicates the wire mesh method produced a measurement that was slightly more accurate than the wooden tray method.

**Fig. 10:**
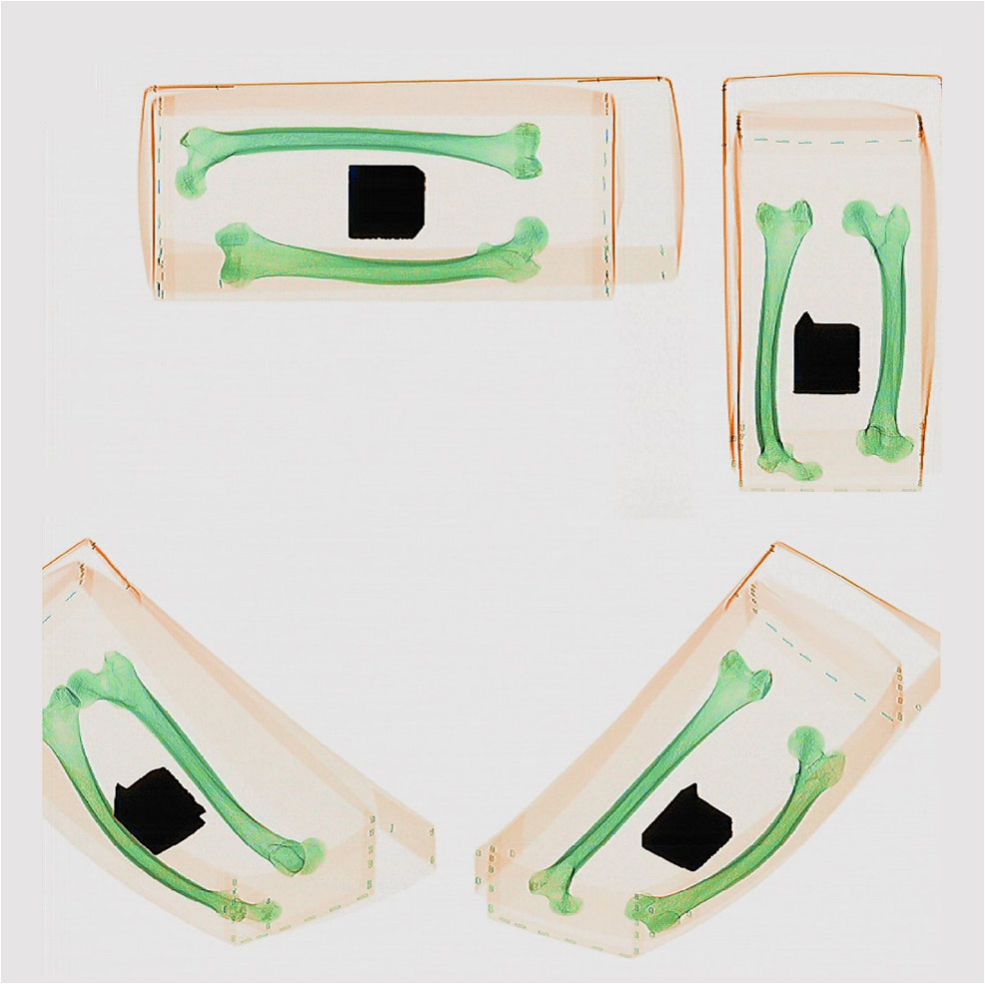
Images of the same two femora in different positions on the scanner belt highlighting the distortion produced in the resulting image

The measurement can be done reasonable accurately if the bone is orientated in the direction of the scanner belt’s travel. The distortion increases significantly once the bone lies at a different angle (Fig. 10), introducing an error in the measurement of the bone’s length. A grid system (the wire mesh method) appears to be the best method to use because once the grid square size is known, it is a matter of counting the number of squares making up the length of the bone and doing the calculation. Even if the squares are distorted in the image, and the bone lying on top of the grid is also distorted, they can still be counted and used because the distortion is the same for the bone and the grid. Therefore, it is expected that with a more accurate grid system, the measurement can be improved.

## Discussion

One of the roles of forensic anthropology in a mass fatality investigation is to assist with the identification of human bone, whether burnt or fragmented amongst other alterations [5, 10, 11]. Forensic anthropologists have to answer questions such as the following: Is this material bone? Is it human? How many individuals are present?. They also undertake where possible a biological profile of the individual (age-at-death, sex, stature, ancestry, and unique identifying features) and reconstruction of the remains to aid in identification [41, 42]. The aim of this study was to address these questions using an imaging method not used before in a MFI and that might be found outside of the usual hospital environment, as imaging of body bags has been recommended for mass fatality incidents prior to their opening [11–16, 19, 20].

The general shape of the objects scanned can be easily seen and therefore identified without having to open the body bag at all. The colour images produced by the baggage scanner allowed differentiation between organic (orange), inorganic (green), and metallic materials (blue). The position of personal effects as well as potentially harmful objects can also be seen, and the image can be used as a guide prior to manual searching of the body bag. Unfortunately, there is little benefit on zooming in on an image because of the comparatively low resolution of the scanner compared with conventional 2D radiography. The lightness of the objects in the body bags for this study meant they had to be supported on thin wooden trays to stop any movement when passing through the lead curtains at the tunnel entrance and introducing image distortion. While there is a risk that the make-up of the wooden tray may have confounded the subsequent analysis and may result in an error if the observer is not aware that it is not part of the body bag contents, we consider this to be unlikely as there was no indication of this in any of our questionnaire responses.

The study examined fresh dead pig tissue as well as archaeological and anatomical (nineteenth century) skeletonised human remains. The fleshed, wet bone is mostly organic and so showed up in shades of orange, with the amount of detail in the bone seen dependant on how thick a layer of flesh is covering the bone. Dry bone shows up in shades of green as it has lost most of its water and organic content. The bones were clearly seen within the range of the image quality, allowing non-human and human bone to be distinguished from each other, as well as identifying when there were body parts belonging to more than one individual in the body bag. These are very important questions that need answering in the initial or triage stages of the identification process. All twelve observers commented on the ease with which they were able to identify the bones and to distinguish between human, non-human, and other miscellaneous objects. They also commented on how the different colours afforded by the materials discrimination aided in this identification significantly, although it did require some training of the eye to pick out the finer details in the images. Several of the observers found that where dealing with this type of image became difficult was when there was an overlapping of remains: this is because the scanner displays *Z*_eff_ and therefore bones of a similar nature, due to the way they have been stored or their age for example, will be displayed with the same shade of colour and therefore cannot be visually separated. In this case, there may be a benefit in reverting to the conventional black and white image that does not offer material discrimination but represents the relative X-ray absorption. This means that details needed to establish a biological profile, identify trauma, and see identifying features such as pathological conditions may not always be possible and that users may occasionally need to use the other imaging modalities offered by these machines, which will increase the demand on operator training.

The observers have stipulated that using baggage scanners to image body bags, when a CT scanner is not available, could be used as a screening tool because it gives a good overview of what is inside the sealed body bag, but not enough detail for a complete biological profile. Performing a biological profile is challenging using this imaging method, especially with skeletonised remains. The possibility of estimating age-at-death and sex relies on the orientation of the remains. In addition, there are several methods for estimating age [37, 38], which would require a much clearer image that can be zoomed into to see detail. Despite this, 83% of the observers identified the juvenile bones in the questionnaire and 100% of observers identified those in the other images as adult. Stature is possible but a mesh method or a reference object of known size is required in order to obtain bone length. A more detailed mesh would be required in order to be more precise if the 12.7-mm squares of the mesh are too large and smaller squares are required. Users should be advised that the reliability of such a measurement depends strongly on the orientation of the bone. All the observers involved in this study commented on the lack of a scale in the images in order to measure the length of the bone to calculate stature from the remains. Simple imaging scaling will only work if the bone is positioned along the direction of the scan. If it is oriented perpendicular to this direction, it will be foreshortened due to the design of the baggage scanner’s detector. Because the degree of foreshortening depends on the position across the belt, using a scaling object will not work and a grid should be used. At other angles, this geometrical effect results in an unnaturally curved bone that indicates reliable length measurement is not possible (Fig. 10).

## Conclusion

Airport baggage scanners are large enough to accommodate a body bag, and are frequently found in private and government buildings in virtually every country around the world. The aims of this study were to determine if the image produced would be clear and detailed enough to view the contents of the body bag from a forensic anthropological perspective working in a mass fatality incident. The skeletal elements present, the minimum number of individuals, whether the bones are human or not, the partial or preliminary assessment of biological profile (e.g. juvenile vs adult) and the identification of some pathological conditions appears to be possible. The presence and location of hazardous objects, projectiles, and personal effects were also identified.

This study was not intended to provide an alternative to a 3D imaging technique such as CT, and using a baggage scanner should only ever be considered if a more sophisticated imaging system is not available. There are a number of limitations to bear in mind when using a baggage scanner. Radiological views are not always anatomical, especially those with overlapping bones that are not easy to separate out. When in materials discrimination mode, the X-ray scanner is able to determine that there is material of a certain *Z*_eff_ in a particular area and this can easily differentiate different types of materials and help classify objects such as fresh/dry bone, bullets, weapons, and personal effects. However, if there are several pieces of similar material lying on top of each other, then the X-ray detector may not be able to separate them and other imaging settings should be investigated. The distortion found in the image needs to be considered before tasks such as bone measurements can be done with accuracy and to the untrained could be misinterpreted as evidence of bowing (osteomalacia), and the image quality may limit the benefit increasing the magnification of an image (image zoom).

This study has shown that this method of imaging has the potential to be very useful in the primary survey and triage stage of imaging done at the beginning of the Disaster Victim Identification process. When considering their response plans, emergency responders should consider including baggage scanners as a contingency for screening body bags. This should be based on a review of the functionality provided by the scanners in a particular geographical location. Further study is needed focusing on different stages of decomposition; imaging full length bodies both with and without soft tissue present; understanding how other functionality within the scanner could help improve image quality; scoring grayscale images; refining the grid system for bone measurement; and correcting the distortion in the images with a software program. The scenarios may include soil, debris, ash, and a larger number of commingled bones. Training of DVI staff in recognising images of human remains in these scanners would also be important (e.g. what cremated bone looks like in a colour image; being aware of bone shape distortion) as provided by the observers’ questionnaires.

## Electronic supplementary material

ESM 1 (PDF 612 kb)

ESM 2 (PDF 182 kb)

ESM 3 (PDF 361 kb)
