# Supplementary material for: Baggage scanners and their use as an imaging resource in mass fatality incidents
Source: Int J Legal Med. 2019 Aug 8;134(4):1419–29. doi: 10.1007/s00414-019-02132-y (PMC7295821; doi:10.1007/s00414-019-02132-y)
Supplement: Supplementary file 1 — (PDF 612 kb) [file 414_2019_2132_MOESM1_ESM.pdf]

Dissertation questionnaire
Genevra D'Arcy
Supervisor: Nick Marquez-Grant

Occupation:

Length of experience:

Scenario:
You have been deployed in your capacity as a forensic anthropologist to assist in a mass fatality incident that has taken place in a remote area of Puntland, Somalia. Your radiology equipment has been detained at the airport in Kenya due to the customs paperwork having gone missing, and that has to be found before it will be released and can join your team in Somalia. There are time constraints on identifying the victims due mainly to religious needs and that diesel for the generators (the only source of electricity) is running low. Your fixer is sourcing more diesel, but it will take a few days to get to your location. The local airport has several working baggage scanners, and these have been pressed into service to start the identification process. The following images have been taken in the triage system and you have been asked to review them from a forensic anthropological perspective before opening the body bags (you are in a hostile environment after all!).

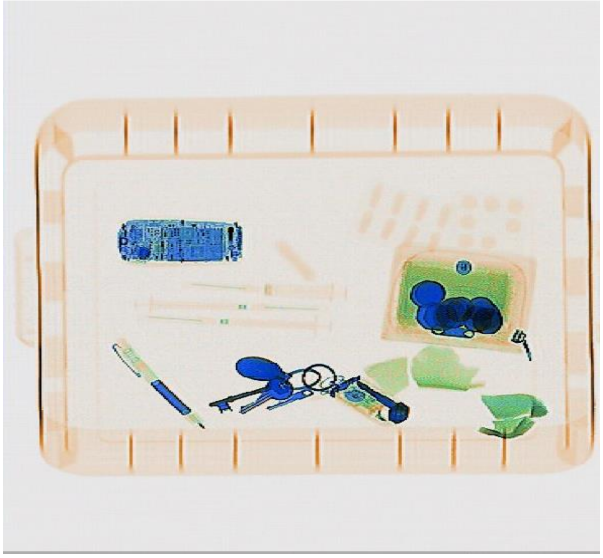

Figure 1.

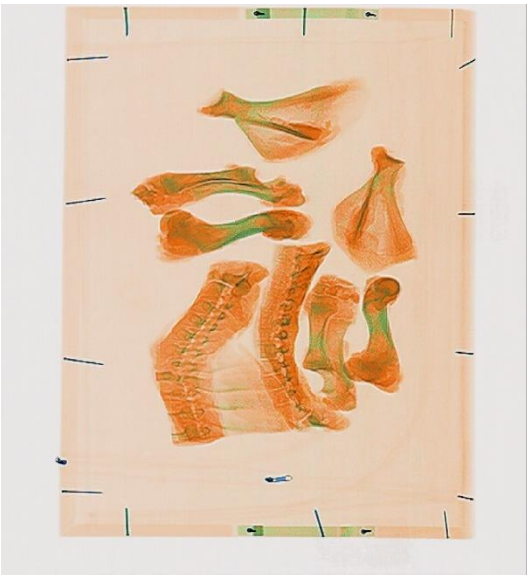

Figure 2:

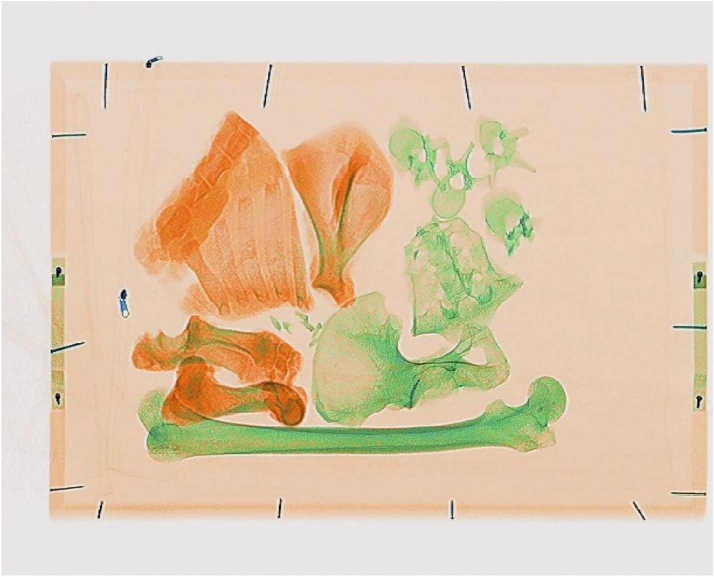

Figure 3:

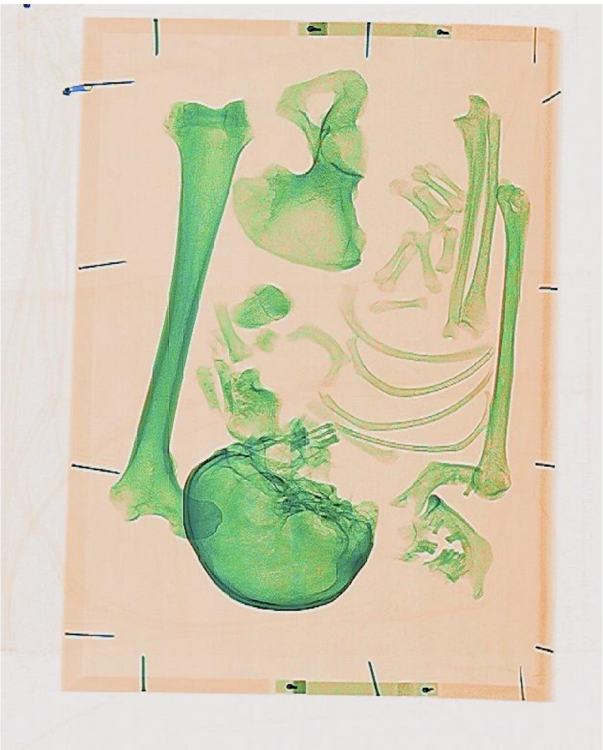

Figure 4:

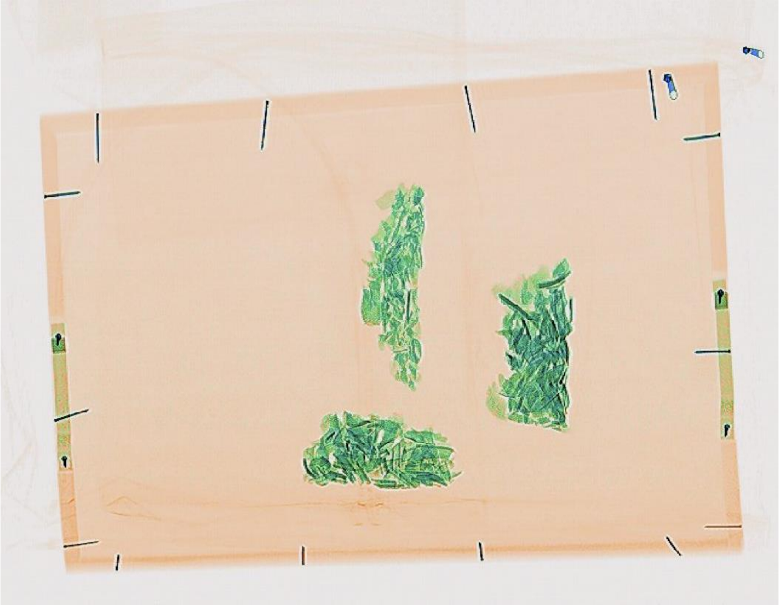

Figure 5:

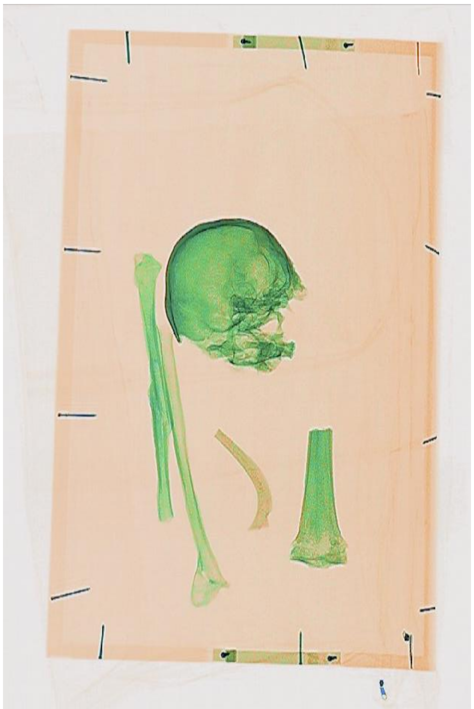

Figure 6:

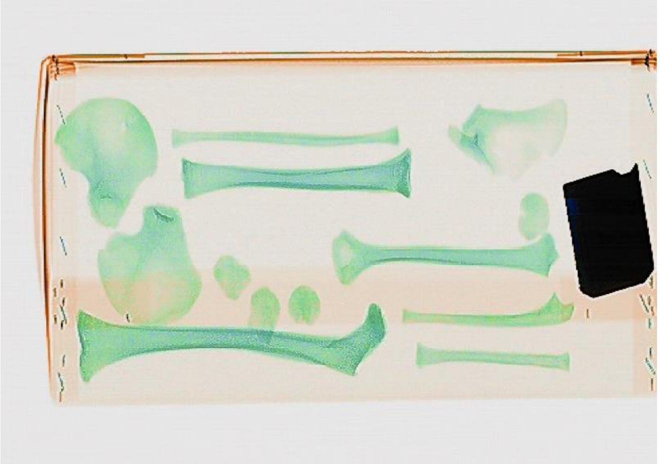

Figure 7:

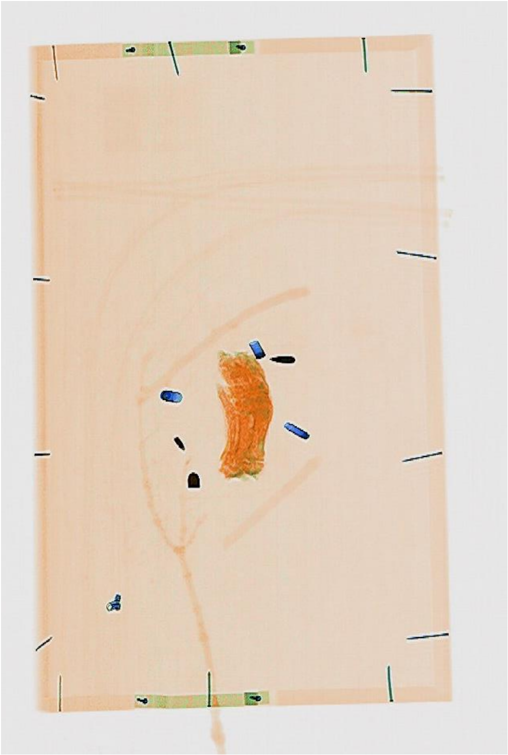

Figure 8:

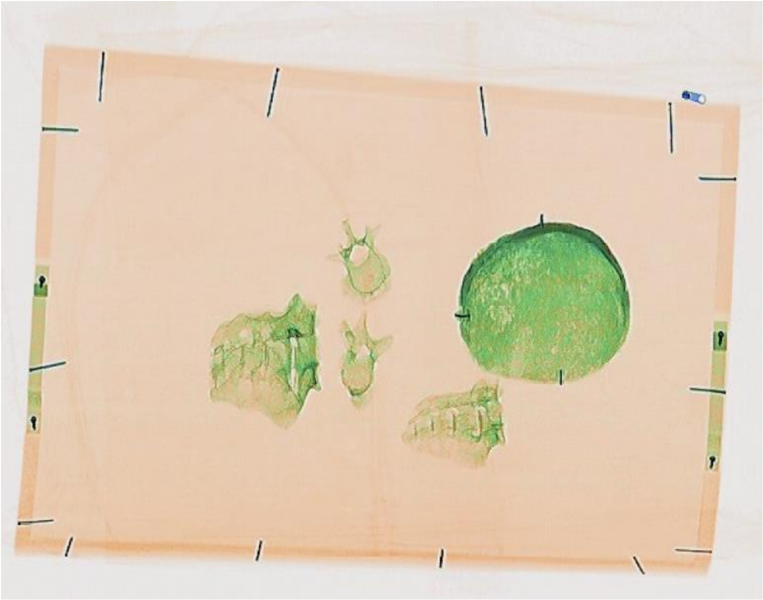

Figure 9:

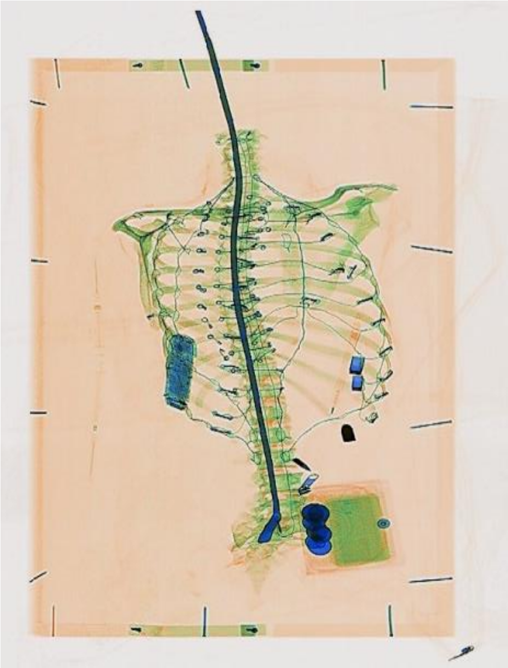

Figure 10:

Please comment on your thoughts about this type of imaging and whether you would be happy to use it when other methods aren't available:
