# Supplementary material for: Baggage scanners and their use as an imaging resource in mass fatality incidents
Source: Int J Legal Med. 2019 Aug 8;134(4):1419–29. doi: 10.1007/s00414-019-02132-y (PMC7295821; doi:10.1007/s00414-019-02132-y)
Supplement: Supplementary file 2 — (PDF 182 kb) [file 414_2019_2132_MOESM2_ESM.pdf]

BAGGAGE SCANNERS AND THEIR USE AS AN IMAGING RESOURCE IN MASS FATALITY INCIDENTS

Genevra D’Arcy, Nicholas Márquez-Grant, David W. Lane

Table 1: Items scanned in groupings to imitate real scenarios with organic and inorganic items mixed together. Bones are either wet and recently defleshed animal bones, or dry human archaeological specimens.

Group	Items	Condition	Image colour
1	pig’s trotters wrapped in clingfilm	Wet, fleshed, frozen then defrosted before imaging	Orange
2	pig’s trotters wrapped in cling film<br>Natural debris (branches, leaves)	Wet, fleshed, not frozen, refrigerated<br>Dry, freshly dropped leaves & sticks	Orange<br>Orange
3	Pig’s trotters wrapped in clingfilm<br>Natural debris (branches, leaves)<br>Metal debris (bullets, casings, needles)	Wet, fleshed refrigerated<br>Dry, freshly dropped<br>Unused bullets, spent casings, new sheathed needles	Orange<br>Orange<br>Blue
4	Pig’s trotters wrapped in clingfilm<br>Natural debris (leaves, branches)<br>Mandible and maxilla fragments<br>Loose teeth	Wet, fleshed refrigerated<br>Dry, freshly dropped<br>Human, archaeological, dry<br>Human, archaeological, dry	Orange<br>Orange<br>Green<br>Green
5	Pig’s trotter wrapped in clingfilm<br>Natural debris (branches, leaves)	Charred for 10 mins in an open fire<br>Dry, freshly dropped	Orange<br>Orange
6	1 x section of spine with ribs<br>1 x section of spine<br>4 x long bones<br>2x scapulae	Fleshed, wet, animal<br>Fleshed, wet, animal<br>Defleshed, wet, animal<br>Fleshed, wet, animal	Orange<br>Orange<br>Orange/green<br>Orange
7	1 x section of spine with ribs<br>2 x long bones<br>1 x scapula<br>1 x femur<br>1 x os coxae<br>1 x sacrum<br>4 x vertebrae<br>6 x loose teeth	Fleshed, wet, animal<br>Defleshed, wet, animal<br>Fleshed, wet, animal<br>Human, archaeological, dry<br>Human, archaeological, dry<br>Human, archaeological, dry<br>Human, archaeological, dry<br>Human, archaeological, dry	Orange<br>Orange<br>Orange<br>Green<br>Green<br>Green<br>Green<br>Green
8	1 x femur<br>1 x os coxae<br>1 x sacrum<br>4 x vertebrae<br>6 x loose teeth<br>3 x ribs<br>2 x large long bones<br>2 x vertebrae<br>2 x small long bones<br>3 x ribs	Human, archaeological, dry<br>Human, archaeological, dry<br>Human, archaeological, dry<br>Human, archaeological, dry<br>Human, archaeological, dry<br>Human, archaeological, dry<br>Animal, recent, dry<br>Animal, recent, dry<br>Animal, recent, dry<br>Animal, recent, dry	Green<br>Green<br>Green<br>Green<br>Green<br>Green<br>Green<br>Green<br>Green<br>Green
9	1 x skull with teeth<br>1 x mandible with teeth<br>8 x vertebrae<br>1 x scapula<br>7 x ribs<br>2 x humerus<br>2 x radius<br>2 x ulna<br>2 x bullets<br>2 x syringes and needles<br>Natural debris<br>T-shirt	Human, archaeological, dry<br>Human, archaeological, dry<br>Human, archaeological, dry<br>Human, archaeological, dry<br>Human, archaeological, dry<br>Human, archaeological, dry<br>Human, archaeological, dry<br>Human, archaeological, dry<br>Unused bullets<br>New sheathed needles<br>Dry, freshly dropped<br>Cotton mix	Green<br>Green<br>Green<br>Green<br>Green<br>Green<br>Green<br>Green<br>Dark blue<br>Orange/Blue<br>Orange<br>Orange
10	2 x os coxae<br>3 x femur<br>2 x bullets<br>2 x casings<br>1 x mobile phone<br>1 x wallet with coins<br>2 x syringes and needles<br>1 x trousers<br>Natural debris	Human, archaeological, dry<br>Human, archaeological, dry<br>Unused bullets<br>Unspent cartridges<br>Smart phone<br>Leather with a zipped compartment<br>New sheathed needles<br>Cotton mix<br>Dry, freshly dropped	Green<br>Green<br>Dark blue<br>Light blue<br>Blue<br>Orange/blue<br>Orange/blue<br>Orange<br>Orange

11	Artificially articulated skeletal torso including sacrum but excluding arms, pelvis, head and legs<br>Mobile phone right shirt pocket<br>Pen left shirt pocket<br>Wallet with coins by left hip area<br>2 x bullets and casings	Human, archaeological, dry<br><br><br>Old-style, Nokia 3310<br>Plastic, ball point<br>Leather with zipped compartment<br>Unused bullets & spent casings	Green<br><br><br>Blue<br>Orange<br>Orange/blue<br>Blue
12	1 x skull with teeth<br>2 x maxilla fragments with teeth<br>2 x mandible fragments & teeth<br>1 x humerus<br>1 x radius<br>1 x ulna<br>1 x femur<br>1 x os coxae<br>3 x tarsals<br>3 x metatarsals<br>2 x distal phalanges<br>9 metacarpals	All bones in this group are human, archaeological and dry.	Green<br>Green<br>Green<br>Green<br>Green<br>Green<br>Green<br>Green<br>Green<br>Green<br>Green<br>Green
13	3 x sachets of fragmented bones	Cremated, human, archaeological, dry	Dark green
14	1 x calotte<br>2 x vertebrae<br>2 x sacrum	Human, archaeological, dry, showing infection lesions<br>Human, archaeological, dry with osteophytosis<br>Human, archaeological, dry, one with spina bifida occulta	Green<br>Green<br>Green
15	1 x skull minus mandible<br>2 x fibula<br><br>1 x rib<br>1 x tibia	Human, archaeological, dry, frontal bone damage<br>Human, archaeological, dry, 1: mal-aligned ante-mortem fracture, 2: damage to epiphysis<br>Animal, recent, dry, cut marks<br>Human, archaeological, dry, proximal third only	Green<br>Green<br><br>Light green<br>Green
16	1 x mobile phone<br>2 x tablet blister strips<br>4 x pieces of glass<br>1 x pen<br>3 x syringes and needles<br>1 x tampon<br>1 x set of keys with car fob<br>1 x wallet	Old-style, Nokia 3310<br>Paracetamol, cold & flu tablets<br>Broken glass, coloured, thick<br>Metal and rubber<br>New, sheathed needles<br>Wrapped, unused<br>Metal, electronic parts<br>Leather with zipped compartment	Blue<br>Orange<br>Green<br>Orange/blue<br>Orange/blue<br>Orange<br>Blue<br>Orange/blue
17	1 x humerus & epiphysis<br>1 x scapula<br>1x radius<br>1 x ulna<br>1 x femur<br>1 x tibia<br>1 x fibula<br>3 x epiphyses<br>2 x ilia<br>1 x lead block	All bones are juvenile human, archaeological and dry<br><br><br><br><br><br><br><br><br>Used as a weight to stop movement	Green<br>Green<br>Green<br>Green<br>Green<br>Green<br>Green<br>Green<br>Green<br>Black
18	3 x os coxae	Human, archaeological, dry	Green
19	2 x os coxae<br><br>1 x lead block	1 x human, archaeological, dry<br>1 x cast<br>Used as a weight to stop movement	Green<br>Orange<br>Black
20	6 x femur<br><br>1 x lead block	Human, archaeological, dry, all of different lengths<br>Used as a weight to stop movement	Green<br><br>Black
21	1 x femur on a<br>wire mesh grid	Human, archaeological, dry<br>wire mesh for measuring	Green<br>Blue
22	2 x femur<br><br>1 x lead block	Human, archaeological, dry<br>Scanned in various positions to highlight distortion<br>Used as a weight to stop movement	Green<br><br>Black
