# Supplementary material for: Baggage scanners and their use as an imaging resource in mass fatality incidents
Source: Int J Legal Med. 2019 Aug 8;134(4):1419–29. doi: 10.1007/s00414-019-02132-y (PMC7295821; doi:10.1007/s00414-019-02132-y)
Supplement: Supplementary file 3 — (PDF 361 kb) [file 414_2019_2132_MOESM3_ESM.pdf]

Genevra D'Arcy, Nicholas Márquez-Grant, David W. Lane

Table 2: Results of the questionnaire. Observers 1 to 12 are listed across the top with their years of experience in forensic anthropology recorded in brackets. "Yes" and "No" answers indicate positive identification of the individual items listed. No biological profiling was done, only identification.

**Observer (experience in years):** →	**1 (17)**	**2 (8)**	**3 (19)**	**4 (6)**	**5 (10)**	**6 (2)**	**7 (6)**	**8 (2)**	**9 (3)**	**10 (15)**	**11 (6)**	**12 (9)**
**Fig. 1**												
Personal effects:												
1x mobile phone	Yes	Yes	Did not list	Yes	Yes	Yes	Yes	Yes	No	Stated as	Stated as	Yes
1x tampon	Yes	No	objects,	No	No	No	No	No	No	no	no	No
2x tablet blister packs	Yes	No	ID'd 2 as	Yes	No	Yes	No	Yes	No	objects	human	Yes
3x syringes and needles	Yes	Yes	bone	Yes	No	Yes	Yes	Yes	Yes	of anthro	remains	Yes
1x wallet with coins	Yes	Yes	fragments	Yes	Yes	Yes	Yes	Yes	Yes	interest	present	Yes
1x pen	Yes	Yes		Yes	Yes	Yes	Yes	No	Yes			Yes
1x set of 3 keys & a car fob	Yes	Yes		Yes	Yes	Yes	Yes	Yes	Yes			Yes
4x pieces of glass	No	No		No	No	Yes	No	No	No			No
**Fig. 2**												
Animal bones:	Yes	Stated	Stated as	Yes	Yes	Yes	Yes	Yes	Yes	Stated as	Yes	Yes
2x scapulae, defleshed, wet	Yes	as	Animal	Yes	Yes	Yes	Yes	Yes	No	non-	Yes	Yes
4x long bones, defleshed, wet	Yes	Animal	bones	Yes	Yes	Yes	Yes	No	No	human	Yes	Yes
1x section of spine with ribs, fleshed, wet	Yes	bones		No	Yes	No	Yes	Yes	No	bones	Yes	Yes
1x section of spine, fleshed, wet	Yes	Yes		No	Yes	Yes	Yes	Yes	Yes		Yes	Yes
**Fig. 3**												
Animal and human bones:	Yes	Yes	Stated as	Yes	Yes	Yes	Yes	Yes	Yes	Stated as	Stated as	Yes
1x scapulae, defleshed, wet	Yes	Yes	Animal	Yes	Yes	Yes	Yes	Yes	No	non-	animal	Yes
2x long bones, defleshed, wet	Yes	Yes	(orange)	Yes	Yes	Yes	Yes	Yes	No	human	bones	Yes
1x section of spine with ribs, fleshed, wet	Yes	Yes	and	Yes	Yes	Yes	Yes	Yes	Yes	bones		Yes
4x vertebrae, human, dry	Yes	Yes	human	Yes	Yes	Yes	Yes	Yes	Yes	Yes	Yes	Yes
1x sacrum, human, dry	Yes	Yes	(green)	Yes	Yes	Yes	Yes	Yes	Yes	Yes	Yes	Yes
1x os coxae, human, dry	Yes	Yes	bones	Yes	Yes	Yes	Yes	Yes	Yes	Yes	Yes	Yes
6x loose teeth, human, dry	Yes	Yes		Yes	Yes	Yes	Yes	Yes	Yes	Yes	No	No
1x femur, human, dry	Yes	Yes		Yes	Yes	Yes	Yes	Yes	Yes	Yes	Yes	Yes
**Fig. 4**												
Commingled bones:												
1x femur, human, dry	Yes	Yes	Stated as	Yes	Yes	Yes	Yes	Yes	Yes	Yes	Yes	Yes
1x os coxae, human, dry	Yes	Yes	human	Yes	Yes	Yes	Yes	Yes	Yes	Yes	Yes	Yes
9x metacarpals, human, dry	Yes	Yes	bones, did	Yes	Yes	Yes	Yes	Yes	Yes	Yes	Yes	Yes
1x radius, human, dry	Yes	Yes	not list	Yes	Yes	Yes	Yes	Yes	Yes	Yes	Yes	Yes
1x ulna, human, dry	Yes	Yes	them	Yes	Yes	Yes	Yes	Yes	Yes	Yes	Yes	Yes

1x humerus, human, dry	Yes	Yes		Yes	Yes	Yes	Yes	Yes	Yes	Yes	Yes	Yes
5x ribs including 1st, human, dry	Yes	Yes		Yes	Yes	Yes	Yes	Yes	Yes	Yes	Yes	Yes
1x clavicle, human, dry	Yes	No		No	No	Yes	No	No	Yes	No	No	No
3x metatarsals, human, dry	Yes	Yes		No	No	No	Yes	No	Yes	No	No	No
2x foot phalanges, human, dry	Yes	No		No	No	No	Yes	Yes	Yes	No	No	No
1x talus, human, dry	Yes	Yes		No	No	Yes	Yes	Yes	Yes	Yes	No	No
1x calcaneus, human, dry	Yes	Yes		No	Yes	Yes	Yes	Yes	Yes	Yes	No	Yes
1x tarsal, human, dry	Yes	Yes		No	No	No	Yes	No	Yes	No	No	No
1x cranium with 5 teeth insitu, human, dry	Yes	Yes		Yes	Yes	Yes	Yes	Yes	Yes	Yes	Yes	Yes
2x mandible fragments with teeth, human, dry	Yes	Yes		Yes	Yes	Yes	Yes	Yes	Yes	Yes	Yes	Yes
2x maxillae fragments with teeth, human, dry	Yes	Yes		No	Yes	No	Yes	Yes	Yes	No	No	No
**Fig. 5**									Bone	Bone	Human	Unable to
Cremated bones, human, dry	Bone frags	No	Bone frags	Yes	Yes	Yes	Yes	No	frags	frags	remains	ID
**Fig. 6**												
Trauma:												
1x cranium with frontal bone damage, human, dry	No	Yes	Stated as	No	Correctly	Yes	Correctly	No	No	Yes	Yes	Identified
1x proximal third of a tibia, human, dry	Yes	Yes	Human	Yes	listed	Yes	listed	Yes	Yes	Yes	Yes	2 bones
1x rib with gnaw and cut marks, animal, dry	Yes	No	bones	No	bones	No	bones	No	No	No	No	but no
1x fibula missing the head, damage to distal	Yes	No	except rib	Frag.	but not	Yes	but not	No	Yes	No	No	trauma
epiphysis, human, dry				bone	trauma		trauma					
1x fibula with healed but misaligned ante-mortem	Yes	Yes	Yes	Frag.		Yes	Yes	Yes	Yes	Yes	Yes	Yes
fracture, human, dry				bone								
**Fig. 7**												
Juvenile human bones:	Yes			Yes	Yes	Yes	Yes	Yes	Yes	Not	Yes	Yes
2x ilia	Yes	Stated	Not	Yes	Yes	Yes	Yes	Yes	Yes	answered	Yes	Yes
1x tibia	Yes	as	answered	Yes	Yes	Yes	Yes	Yes	Yes		Yes	Yes
1x fibula	Yes	Juvenile		Yes	Yes	Yes	Yes	Yes	Yes		Yes	Yes
1x scapula	Yes	bones,		Yes	Yes	Yes	Yes	Yes	Yes		Yes	Yes
1x humerus	Yes	no		Yes	Yes	Yes	Yes	Yes	Yes		Yes	Yes
1x humeral head	No	specifics		Yes	Yes	Yes	Yes	No	Yes		Yes	No
1x ulna	Yes			Yes	Yes	Yes	Yes	Yes	Yes		Yes	Yes
1x radius	Yes			Yes	Yes	Yes	Yes	Yes	Yes		Yes	Yes
1x femur	Yes			Yes	Yes	Yes	Yes	Yes	Yes		Yes	Yes
1x femoral head	Yes			Yes	Yes	Yes	Yes	Yes	Yes		Yes	No
2x unfused epiphyses (tibia & femur)	No			No	No	Yes	Yes	No	Yes		Yes	No
**Fig. 8**												
(Charred) pig trotter	Yes	Yes	Yes	Stated as	Yes	Yes	Yes	No	No	Yes	Stated as	Non-
Branches	No	No	No	no	No	No	No	Yes	No	No	no	human
Bullets (projectiles) and casings (cartridges)	Yes	No	No	human	Yes	Yes	No	Yes	Yes	Yes	human	hand
				remains							remains	
**Fig. 9**												

Pathology:<br>1x calotte with infection lesions (caries sicca)<br>1x lumbar vertebra with osteophytosis<br>1x thoracic vertebra with osteophytosis<br>1x sacrum with age-related wearing<br>1x unfused sacrum (spina bifida occulta)	Yes<br>Yes<br>Yes<br>Yes<br>Yes<br>Yes	No<br>Correctly<br>listed<br>bones<br>but not<br>path.	No<br>Stated as<br>human<br>bones only	No<br>Correctly<br>listed<br>bones<br>but not<br>pathology	No<br>Correctly<br>listed<br>bones<br>but not<br>pathology	Yes<br>Yes<br>Yes<br>Yes<br>Yes<br>Yes	Some<br>Correctly<br>listed<br>bones<br>but not<br>pathology	No<br>Correctly<br>listed<br>bones<br>but not<br>pathology	Some<br>Yes<br>No<br>No<br>No<br>No	Some<br>No<br>No<br>No<br>No<br>Yes	No<br>Correctly<br>listed<br>bones<br>but not<br>pathology	No<br>Correctly<br>listed<br>bones<br>but not<br>pathology
**Fig. 10**<br>Dressed, articulated human torso minus arm and leg bones, os coxae<br>Rod, screws and wires used to articulate the bones<br>Mobile phone in right shirt pocket<br>Pen in left shirt pocket<br>Wallet near hip area<br>2x bullets and casings under left side of torso	 Yes<br><br>Yes<br><br>Yes<br>No<br>Yes<br>Yes	 Yes<br><br>No<br><br>Yes<br>No<br>Yes<br>No	 Stated as<br>human<br>anatomical<br>model<br>bones	 Yes<br><br>No<br><br>Yes<br>No<br>Yes<br>No	 Yes<br><br>Yes<br><br>Yes<br>No<br>Yes<br>Yes	 Yes<br><br>Yes<br><br>Yes<br>No<br>Yes<br>Yes	 Stated as<br>teaching<br>skeleton<br>with<br>other<br>objects	 Yes<br><br>No<br><br>Yes<br>Yes<br>Yes<br>Yes	 Yes<br><br>No<br><br>No<br>No<br>No<br>No	 Yes<br><br>No<br><br>No<br>No<br>No<br>Yes	 Yes<br><br>No<br><br>No<br>No<br>No<br>No	 Yes<br><br>No<br><br>No<br>No<br>Yes<br>No
